# Label propagation-based semi-supervised feature selection on decoding clinical phenotypes with RNA-seq data

**DOI:** 10.1186/s12920-021-00985-0

**Published:** 2021-08-31

**Authors:** Xue Jiang, Miao Chen, Weichen Song, Guan Ning Lin

**Affiliations:** 1 Shanghai Mental Health Center, Shanghai Jiao Tong University School of Medicine, School of Biomedical Engineering, Shanghai Jiao Tong University, Shanghai, 200030 China; 2 Shanghai Key Laboratory of Psychotic Disorders, Shanghai, 200030 China

**Keywords:** Biomarkers that corresponding to clinical phenotypes, Label propagation clustering, Feature selection

## Abstract

**Background:**

Clinically, behavior, cognitive, and mental functions are affected during the neurodegenerative disease progression. To date, the molecular pathogenesis of these complex disease is still unclear. With the rapid development of sequencing technologies, it is possible to delicately decode the molecular mechanisms corresponding to different clinical phenotypes at the genome-wide transcriptomic level using computational methods. Our previous studies have shown that it is difficult to distinguish disease genes from non-disease genes. Therefore, to precisely explore the molecular pathogenesis under complex clinical phenotypes, it is better to identify biomarkers corresponding to different disease stages or clinical phenotypes. So, in this study, we designed a label propagation-based semi-supervised feature selection approach (LPFS) to prioritize disease-associated genes corresponding to different disease stages or clinical phenotypes.

**Methods:**

In this study, we pioneering put label propagation clustering and feature selection into one framework and proposed label propagation-based semi-supervised feature selection approach. LPFS prioritizes disease genes related to different disease stages or phenotypes through the alternative iteration of label propagation clustering based on sample network and feature selection with gene expression profiles. Then the GO and KEGG pathway enrichment analysis were carried as well as the gene functional analysis to explore molecular mechanisms of specific disease phenotypes, thus to decode the changes in individual behavioral and mental characteristics during neurodegenerative disease progression.

**Results:**

Large amounts of experiments were conducted to verify the performance of LPFS with Huntington’s gene expression data. Experimental results shown that LPFS performs better in comparison with the-state-of-art methods. GO and KEGG enrichment analysis of key gene sets shown that TGF-beta signaling pathway, cytokine-cytokine receptor interaction, immune response, and inflammatory response were gradually affected during the Huntington’s disease progression. In addition, we found that the expression of SLC4A11, ZFP474, AMBP, TOP2A, PBK, CCDC33, APSL, DLGAP5, and Al662270 changed seriously by the development of the disease.

**Conclusions:**

In this study, we designed a label propagation-based semi-supervised feature selection model to precisely selected key genes of different disease phenotypes. We conducted experiments using the model with Huntington’s disease mice gene expression data to decode the mechanisms of it. We found many cell types, including astrocyte, microglia, and GABAergic neuron, could be involved in the pathological process.

## Background

Neurodegenerative disease is a type of chronic progressive disease with complex pathogenic mechanisms caused by neuronal degeneration, leading to abnormal behavior, mental dysfunction and ultimately death [1–3]. Motor ability, cognitive ability, memory ability and other functions are gradually impaired during the disease progression [4, 5]. It has been reported that there are many pathogenic factors of neurodegenerative disease, such as neurotrophasthenia, impairments of axon transmission, impairments of metabolic pathways, protein misfolding, inflammation, and intestinal microorganism [6–9]. However, single pathogenic factor cannot fully explain the pathogenesis of the disorder. The pathogenesis is still not well understood, and there is no effective treatment for it.

Meanwhile, Huntington’s disease (HD) is a representative neurodegenerative disease, which is caused by a triplet (CAG) repeat elongation in huntingtin (HTT) gene on chromosome 4 that codes for polyglutamine in the huntingtin protein [10]. The mutant protein can enter the nucleus and alter gene transcription [11]. With the accumulation of the mutant protein, numerous interactions between molecules and pathways can be affected, resulting in neuronal dysfunction and degeneration [12, 13]. With the connections between neurons get sparse, the neurons finally died during the disease deterioration, and the volume of striatum tissue decreased markedly [14]. Clinically, motor ability, cognitive, and mental functions are gradually affected.

With the rapid development of high-throughput sequencing technology, large amounts of omics data and biomedical data have been accumulated, providing both opportunities and challenges to develop computational methods for mining biomarkers, such as functional elements and locus in DNA sequences. Further decoding regulatory relationships of those biomarkers to clinical phenotypes is helpful for understanding physiopathologic mechanisms under the abnormal behavior, promoting early diagnosis and interventional treatment for neurodegenerative disease.

Generally, at the transcriptomic level, researchers select key genes affected by diseases based on the hypothesis that disease genes tend to differentially expressed between case samples and normal samples. Nevertheless, the relationship between genes and their functions is complex and multifaceted, namely the same gene can play a role in many different functions. In living organisms, genes interact with each other to produce high-level biological functions, such as motor ability, cognitive ability, memory, emotion, etc. It has been well established that genes that have synergistic effects usually have similar expression patterns, and participate in a same biochemical reaction or in a same pathway [15]. Therefore, searching for gene clusters that are severely affected, and analyzing the biological pathways involved in can be helpful to understand the dynamic molecular process during the degeneration of the disease. The screened key genes and pathways can further be used to decode molecular mechanisms related to clinical abnormal behaviors.

Because of the critical of some essential genes, the annotation of many genes that maintain the normal function of central nervous system is still unclear [16]. Besides, our previous studies shown that the expression level of most lethal phenotype genes are not significantly changed during Huntington’s disease degeneration [17, 18]. Therefore, traditional statistical-based differentially expressed gene selection methods can not effectively select clinical phenotype associated genes for complex neurodegenerative disease. Nevertheless, clustering algorithms often used to detect gene modules. Genes that belong to a same module would have similar function or expression pattern, while genes that belong to different modules usually have very different properties. Moreover, we can use clustering methods to detect high-order biological signals, deepen the understanding of biological process which are seriously affected by the disease.

Based on the objects to be clustered, clustering algorithms can be classified into three categories: gene-based clustering, sample-based clustering, and bi-clustering [19, 20]. Gene-based clustering methods classify the genes with similar expression patterns into one category, such as label propagation algorithm [21], and fuzzy clustering algorithm [22], etc., to get meaningful gene modules. Sample-based clustering methods take the samples as cluster objects, and gene expression is seen as a feature of the sample, which can be used to measure and identify the subtypes of patients. Supervised machine learning technology are often used to conduct cluster analysis of samples. Bi-clustering algorithms cluster genes and samples at the same time, mining genes with similar expression patterns, and further exploring the dynamic changes of gene module function under different sample states [23–25]. Since the function of clustered gene module can be seen as high order biological signal, bi-clustering algorithms are usually used to analyze the changes of biological process during disease degeneration [26].

Meanwhile, label propagation clustering algorithm is a graph-based semi-supervised machine learning method. It is based on guilt-by-association to predict the label information of unlabeled nodes with a few labeled nodes [21]. When the labels of the nodes in the network tend to be stable, the nodes with the same label identity are divided into a same category. Since it is costly to make tags of the samples for big biomedical data, unsupervised and semi-supervised methods have great prospect in this type of applications. According to the above discussion, to identify key genes which could be matched to the complex clinical phenotypes of different disease stages, we designed a semi-supervised feature selection method based on label propagation clustering algorithm (LPFS). LPFS includes two parts: one part is label propagation clustering based on the sample network which is constructed with gene expression data, the other part is the feature selection process based on the feature selection matrix. By conducting alternative iteration of the two steps, we select key genes which could be matched to the complex clinical phenotypes of different disease stages. To our best knowledge, this is the first time to put gene selection and sample clustering into one framework to prioritize disease genes.

To investigate the effectiveness of the biomarkers selected by the LPFS, we also conducted experiments with DESeq2 [27], edgeR [28], limma [29], t-test [30], fold change method (FC) [30], joint non-negative matrix factorization meta-analysis method (jNMFMA) [31], and flexible non-negative matrix factorization method (FNMF) [18]. Finally, we performed GO and KEGG pathways enrichment analysis of key genes identified by LPFS, to explore the affected gene functions underlying the complex clinical phenotypes, gaining a deep understand of the dynamic molecular mechanisms during the disease progression.

The rest of this paper is organized as follows: In “Methods” section, we present the proposed LPFS in detail. In “Results and discussion” section, we illustrate experiments of different methods with RNA-seq data of Huntington’s disease. The enrichment analysis of key genes obtained by LPFS are performed and reported. And the overall discussion of experimental results of various methods are also reported. In “Conclusions” section, conclusions are presented.

## Methods

In this section, we present LPFS approach in detail and discuss the parameter setting of it.

### Label-propagation based semi-supervised feature selection

The gene expression data is denoted as $X=[x_{ij}]_{n \times m}$, where $x_{ij}$ represents the expression level of gene *j* in sample *i*, $x_{i \cdot }$ denotes sample *i*, and $x_{\cdot j}$ denotes gene *j*. $L=\{1, \ldots , c\}$ represents the set of labels, *c* is the number of cluster number, and $l_{i}$ is the label for sample $x_{i \cdot }$, $l_{i} \in L$. The initial category label matrix is denoted as $Y = [y_{ij}]_{n \times c}$, where

1 $$\begin{aligned} y_{ij} = {\left\{ \begin{array}{ll} 1, &\quad \mathrm{if}~l_{i} = j,\\ 0, &\quad \mathrm{otherwise}. \end{array}\right. } \end{aligned}$$

$Y_{i}$ is the *i*-th row in matrix *Y*, representing the initial category label of sample $x_{i \cdot }$.

*H* denotes a vector function $H: X \to R^{c}$. $x_{i \cdot }$ corresponds to a $H_{i}$. $H = [H_{1}^{T}, \ldots , H_{n}^{T}]$ is a $n \times c$ clustering indicator matrix. The category label of $x_{i \cdot }$ is $l_i = arg max_{j \le c}h_{ij}$. $F= [f_{ij}]_{m \times c}$ is feature selection matrix. In this study, we define $||A||_{F} = \root \of {\sum _{i}\sum _{j}a_{ij}^{2}}$, and the $l_{2,1}$ of matrix *A* is $||A||_{2,1} = \sum _{j}(\root \of {\sum _{i}a_{ij}^{2}})$.

To make precision diagnosis of a patient, one key point is to identify biomarkers corresponding to the illness state of the patient correctly. To address the problem, we designed a feature selection method based on label propagation clustering namely, LPFS. LPFS conduct key gene selection during the sample clustering process, filter out redundant features, and select key genes that would well represent and distinguish different category samples. The selected genes should make the sample distance within one class close, and the sample distance between classes farther. Biologically, to identify severely affected genes corresponding to different clinical stages or phenotypes, it is important to select key genes that can distinguish different stages of the disease. Since not all genes have positively contribute to sample classification, therefore, we put $l_{2,1}$ constraint on feature selection matrix to sparse each column of it and filter out noise factors [32]. According to mathematical meaning, LPFS can be formulated as the following optimization problem:

2 $$\begin{aligned}&min_{(H,F)} \quad\sum _{i,j=1}^{n}w_{ij}||\frac{1}{\sqrt{d_{ii}}}H_{i} - \frac{1}{\sqrt{d_{jj}}}H_{j}|| \\ &\quad +\mu \sum _{i=1}^{n}||H_{i}-Y_{i}||^{2} + ||XF-H||_{F}^{2} + \beta ||F||_{2,1} \end{aligned}$$

Here, $\mu$ and $\beta$ are hyper-parameters. The parameter $\mu$ balances the importance of the final label and the initial label of a node during label propagation. The parameter $\beta$ constraints the sparse penalty on the feature selection matrix. $\mu , \beta \in (0,1)$. It should be noted that $||F||_{2,1} = \sum _{j}^{c}(\root \of {\sum _{i=1}^{m}f_{ij}^{2}})$. There only cluster indicator matrix *H* is unknown by fixing *F* in the first three terms of Eq. (2), and there only feature selection matrix *F* is unknown by fixing *H* in the last two terms of Eq. (2). So, we compute the solution for the LPFS via an iterative updating algorithm that alternatively updates *H* and *F*. The detailed solving processes are shown below.

*Step 1*. Define an undirected graph $G=(V,E)$ using gene expression data *X*. We use Gaussian kernel function to measure the relationship between two nodes. The weight matrix of *G* is $W=[w_{ij}]_{n \times n}$, where

3 $$\begin{aligned} w_{ij} = {\left\{ \begin{array}{ll} exp(-||x_{i \cdot } - x_{j \cdot }||^{2}/(2 \delta ^{2})), &\quad \mathrm{if}~i \ne j,\\ 0, &\quad \mathrm{otherwise}. \end{array}\right. } \end{aligned}$$

*Step 2*. Normalize the weight matrix. Let $D=diag\{d_{ii}\}$, where $d_{ii} = \sum _{j=1}^{n}w_{ij}$. Therefore the normalized weight matrix is

4 $$\begin{aligned} Z = D^{-1/2}WD^{-1/2}. \end{aligned}$$

*Step 3*. Initialize the initial category label matrix *Y*, and initialize cluster indicator matrix *H* to *Y*.

*Step 4*. According to the last two terms in Eq. (2), we solve feature selection matrix *F*

5 $$\begin{aligned} min_{(F)} \quad ||XF-H||_{F}^{2} + \beta ||F||_{2,1}. \end{aligned}$$

In this study, each row in the feature selection matrix *F* is randomly initialized in (0, 1). The elements in *F* should be non-negative to keep the contribution of genes not be systematically offset. $\phi _{ij}$ is the Lagrangian multiplier of $f_{ij} \ge 0$. So, we can construct Lagrangian function as below:

6 $$\begin{aligned} L(F) &=\alpha Tr(XFF^{T}X^{T} - 2XFH^{T} + HH^{T}) \\ &\quad + \beta Tr(FUF^{T}) + Tr( \Psi F^{T}). \end{aligned}$$

Here, $U = diag(\frac{1}{2||F^{1}||_{2}}, \ldots , \frac{1}{2||F^{c}||_{2}})$ is an Auxiliary matrix, and $F^{i}$ denotes the *i*-th column of matrix *F*, $\Psi = [\psi _{ij}]$.

The derivation of *F* is

7 $$\begin{aligned} \frac{\partial L}{\partial F} = 2X^{T}XF - 2X^{T}H + 2\beta FU +\Psi . \end{aligned}$$

Based on the KKT condition $\psi _{ij}f_{ij} = 0$, we can get

8 $$\begin{aligned} \frac{\partial L}{\partial F} = 2X^{T}XF - 2X^{T}H + 2\beta FU +\Psi = 0. \end{aligned}$$

9 $$\begin{aligned} (2X^{T}XF - 2X^{T}H + 2\beta FU)_{ij}f_{ij} = -\psi _{ij}f_{ij} = 0. \end{aligned}$$

Equation (9) can be written as

10 $$\begin{aligned} (X^{T}H)_{ij}f_{ij} = (X^{T}XF + \beta FU)_{ij}f_{ij}. \end{aligned}$$

Then, we can get the update role of *F*

11 $$\begin{aligned} f_{ij} \leftarrow f_{ij}\frac{(X^{T}H)_{ij}}{(X^{T}XF + \beta FU)_{ij}}. \end{aligned}$$

*Step 5*. According to the first three terms in Eq. (2), we solve cluster indicator matrix *H*.

12 $$\begin{aligned}Q(H) &= min_{(H)} ~~\sum _{i,j=1}^{n}w_{ij} \left\| {\frac{1}{{\sqrt{d_{{ii}} } }}H_{i} - \frac{1}{{\sqrt{d_{{jj}} } }}H_{j} } \right\| \\ &\quad +\mu \sum _{i=1}^{n}||H_{i}-Y_{i}||^{2} + ||XF-H||_{F}^{2}. \end{aligned}$$

Equation (12) is a convex function. The derivation of *H* is

13 $$\begin{aligned} \frac{\partial Q}{\partial H} = 2[(H - ZH) + 2\mu (H-Y)] - 2(XF - H). \end{aligned}$$

We can get the global optimal solution at the stationary point.

14 $$\begin{aligned}{}[(H - ZH) + \mu (H-Y)] - (XF - H) = 0. \end{aligned}$$

15 $$\begin{aligned} H^{*} = ((1+\mu )I-Z)^{-1}(\mu Y + XF). \end{aligned}$$

The category of sample *i* is

16 $$\begin{aligned} l_{i} = argmax_{j \le c} h_{ij}^{*}. \end{aligned}$$

Therefore, we update the cluster indicator matrix $H=[h_{ij}]_{n \times c}$, where

17 $$\begin{aligned} h_{ij} = {\left\{ \begin{array}{ll} 1, &\quad \mathrm{if}~l_{i} = j,\\ 0, &\quad \mathrm{otherwise}. \end{array}\right. } \end{aligned}$$

*Step 6*. Repeat Step 4 until the objective function of Eq. (5) converges. Then we can get the feature selection matrix *F*.

*Step 7*. Repeat Step 5 until the objective function of Eq. (12) converges. At this point, we obtain the cluster indicator matrix *H*.

*Step 8*. Conduct loop iteration of Step 3 to Step 7, until the objective function of Eq. (2) converges. At this points, we get both the feature selection matrix *F* and cluster indicator matrix *H*.

*Step 9*. Based on rank-product method [30], we calculate the element fluctuation of each row in the feature selection matrix. If the elements in *k*-th row fluctuate significantly, the rank-product value of that row is larger, representing that the corresponding feature gene *k* has a stronger ability to distinguish samples of different categories.

Sorting the rank-product value of each row of the feature selection matrix in descending order, high ranking rows are reserved and low ranking rows are removed from the feature selection matrix.

Low ranking row indicates the elements in that row change very little through different columns, i.e. the corresponding gene has no discrimination ability of different category samples. Therefore, to improve prediction precision and reduce computational complexity, we filter out low ranking genes to conduct next iteration.

*Step 10*. Repeat aforementioned steps from Step 1 to Step 9.

Let function $top(v_{s})$ represents the *s* larger elements of vector *v*.

Since greater elements in $F^{j}$ contribute more to the identification of the specific category *j*, the genes, whose column number in the gene expression matrix equals to the row number of the greater elements in the feature selection matrix, are seen as key features of category *j*, i.e., the genes that could be severely affected under this condition.

In this study, we use $key_{j}$ to denote the key gene set for category *j*.

18 $$\begin{aligned} key_{j} = arg_{i\le m} top_{s} f_{ij}. \end{aligned}$$

The detailed process of LPFS is summarized in Algorithm 1.
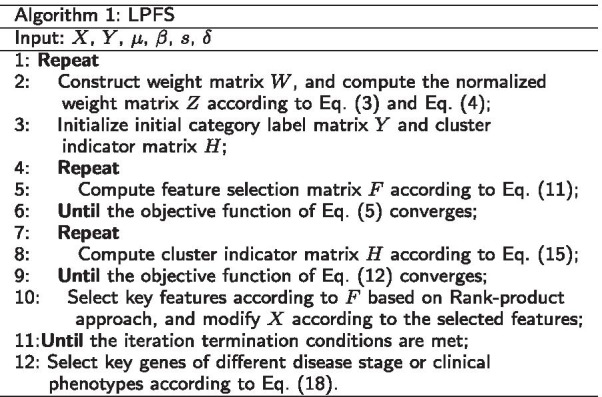


It should be noted that when the number of features is too large, it becomes hard to distinguish connections between samples and to detect modules on sample network. It is difficult to get accurate clustering results since there is no obvious clustering patterns, resulting in unstable and invalid key gene sets. Besides, when the number of clusters is less than the categories, i.e., samples belong to two categories are classified into one cluster in experimental results, it will result in one column of the cluster indicator matrix to be 0. Then, some columns in the feature selection matrix will be all equal to 0, eventually leading to instability of the solution.

Theoretically, the computational process tends to stable as the number of features decreases. In addition, increase the number of samples is helpful to clarify the module structure in the network.

To ensure the convergence of Eq. (2), we first solve feature selection matrix, and then solve cluster indicator matrix. Through the alternative iteration strategy, the Eq. (2) can be convergent to a stable solution. According to experience and suggestions in paper [33], we set $\mu = 0.2$, and $\beta = 0.2$. Besides, we set $\delta = 200$ to ensure $||x_i - x_j||^2/(2 \delta ^2) \in (0,1)$, to get a reasonable connection between genes. In each iteration, low ranking 1000 genes are removed to modify the gene expression data for next iteration. To accurately prioritize the clinical phenotype related genes, 5 iterations were conducted to end the process. Finally, 100 times of the total process were run to get statistical significant result.

## Results and discussion

First, we briefly introduced the gene expression dataset of Huntington’s disease. Second, we demonstrated the experimental results of LPFS. Then, to verify the effectiveness of LPFS, we also conducted experiments with DESeq2, edgeR, limma, t-test, FC, jNMFMA, and FNMF. We further analyzed and discussed the disease gene prediction accuracy of different methods. Finally, we conducted GO and KEGG pathway enrichment analysis of the selected key genes, thus to get a deep understanding of the pathological mechanisms under complex clinical phenotypes of different disease phenotypes.

### Gene expression data

**Fig. 1:**
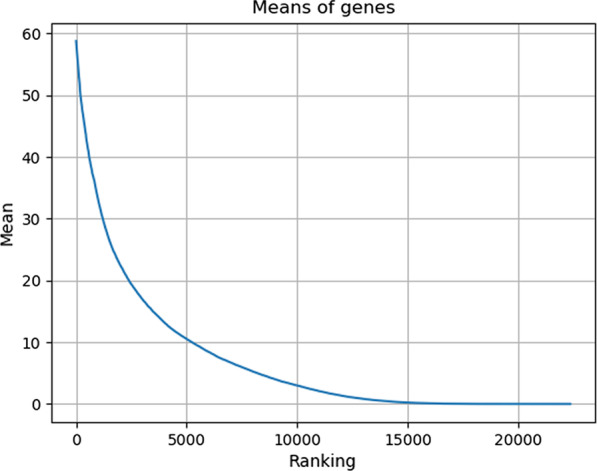
Ranking of the means of gene expression values in all samples

**Fig. 2:**
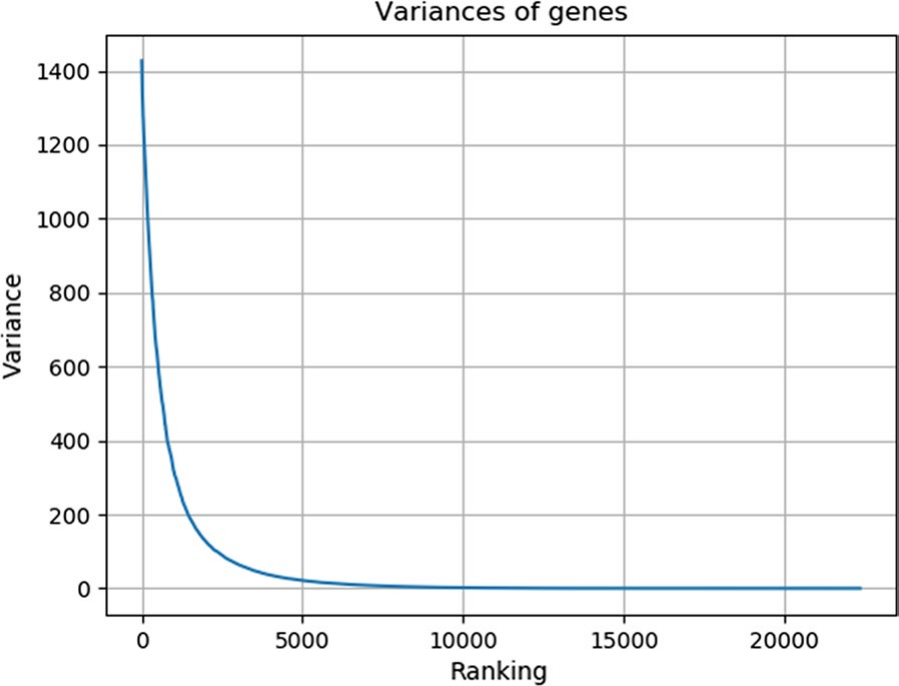
Ranking of the variances of gene expression values in all samples

The gene expression data were downloaded from http://www.hdinhd.org, which were obtained from the striatum tissue of Huntington’s disease mice through RNA-seq technology. The experimental mice in this data set are of 2-month-old, 6-month-old, and 10-month-old. The genotypes include ploy Q20, poly Q80, poly Q92, poly Q111, poly Q140, and poly Q175. The ploy Q20 is normal one, while the rest genotypes are disease ones. There are 16 2-month-old mice of ploy Q20, 16 10-month-old mice of ploy Q20, and 8 mice for other genotypes at each age. The data set contain 23,351 genes. Since the genes expressed robustly across all samples have little contribution to sample classification, we selected top 6000 genes based on the mean (Fig. 1) and variance (Fig. 2) of gene expression data to reduce computational complexity. Besides, to test the accuracy of the selected genes by different methods, we collected 520 modifier genes from the literature [34], including 89 disease genes and 431 non-disease genes. The detailed information of the data set is illustrated in Table 1.

**Table 1:** Gene expression data of Huntington’s disease mice

Tissue	Striatum		
Age	2-Month-old	6-Month-old	10-Month-old
Genotype	Poly Q20	Poly Q80	Poly Q92
	Poly Q111	Poly Q140	Poly Q175

### Prediction performance of LPFS

**Fig. 3:**
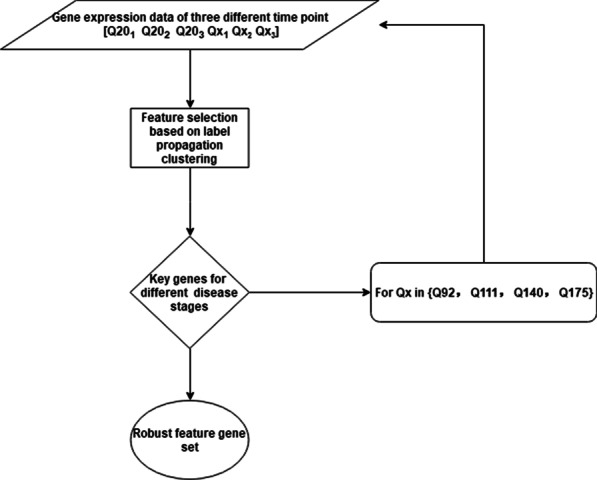
The flowchart of LPFS with Huntington’s disease RNA-seq data

To get robust gene sets of different disease stage, we designed the following experimental pipeline, see Fig. 3. First, we used normal samples with genotype of ploy Q20 under 3 different time points and case samples with genotype of ploy $Q_{x}$ under 3 different time points, $Q_{x} \in \{Q80, Q92, Q111, Q140, Q175\}$, to conduct LPFS. Samples of a genotype at a time point belonged to one category. Thus, there are 6 categories in each experiment. Finally, we ranked genes in descending order according to the elements in each column of the feature selection matrix. Top ranking genes are seen as the key gene set for each category. During the label-propagation based feature selection process, low ranking 1000 genes were removed out from the original gene expression matrix. 5 times iteration have been conducted in each experiment. Finally, 1375 genes were left for each category. To get a robust key gene set, we run each experiment for 100 times. Then, through the intersection of 100 key gene sets for each category, genes that appeared more than 50 times were selected as the key genes for that category. The number of key genes for each category is shown in Table 2.

**Table 2:** The number of key genes for each category

	Normal samples			Case samples		
	2-Month-old	6-Month-old	10-Month-old	2-Month-old	6-Month-old	10-Month-old
Num.	133	73	101	38	22	30

The selected key gene set for each category could be used to describe functional changes during the development of the disease. In summary, we selected 397 marker genes, including 133, 73, 101 specific marker genes for 2-month-old, 6-month-old, 10-month-old normal mice, and 38, 22, 30 specific marker genes for 2-month-old, 6-month-old, 10-month-old case mice, respectively.

The GO and KEGG pathway enrichment analysis of those key gene sets help to get a deep understanding of the intermediate phenotypes and molecular activity of different disease stages [35, 36]. We conducted enrichment analysis with Metascape [37]. The enrichment analysis results for specific marker genes are shown in Tables 3 and 4. From Table 3, we can see that the functions, such as pituitary gland development, aromatic amino acid family metabolic process, arachidonic acid metabolic process, and regulation of adaptive immune response, change greatly during the growth process. From Table 4, we can see that the functions, such as sensory perception of sound, aging, positive regulation of NF-kappaB transcription factor activity, negative regulation of inflammatory response are affected during the disease degeneration.

**Table 3:** The GO and KEGG pathway enrichment analysis of normal mice marker genes by LPFS

GO	Category	Description	Log10(P)
*2-Month-old*			
R-MMU-176412	Reactome Gene Sets	Phosphorylation of the APC/C	− 4.11
GO:0021983	GO Biological Processes	Pituitary gland development	− 3.16
GO:0022412	GO Biological Processes	Cellular process involved in reproduction in multicellular organism	− 3.11
R-MMU-500792	Reactome Gene Sets	GPCR ligand binding	− 2.78
R-MMU-2980736	Reactome Gene Sets	Peptide hormone metabolism	− 2.60
GO:0097305	GO Biological Processes	Response to alcohol	− 2.55
*6-Month-old*			
R-MMU-500792	Reactome Gene Sets	Aromatic amino acid family metabolic process	− 3.64
GO:0048589	GO Biological Processes	Steroid hormone biosynthesis	− 2.05
*10-Month-old*			
GO:0009072	GO Biological Processes	Arachidonic acid metabolic process	− 6.52
mmu00140	KEGG Pathway	Steroid hormone biosynthesis	− 4.54
GO:0019369	GO Biological Processes	Arachidonic acid metabolic process	− 3.97
GO:0002819	GO Biological Processes	Regulation of adaptive immune response	− 3.60
mmu04610	KEGG Pathway	Complement and coagulation cascades	− 3.35
GO:0001580	GO Biological Processes	Response to alcohol	− 3.02
R-MMU-174824	Reactome Gene Sets	Response to alcohol	− 2.76
GO:0010466	GO Biological Processes	Response to alcohol	− 2.50

**Table 4:** The GO and KEGG pathway enrichment analysis of case mice marker genes by LPFS

GO	Category	Description	Log10(P)
*2-Month-old*			
GO:0007605	GO Biological Processes	Sensory perception of sound	− 2.63
*6-Month-old*			
GO:0002021	GO Biological Processes	Response to dietary excess	− 3.84
R-MMU-2559586	Reactome Gene Sets	DNA Damage/Telomere Stress Induced Senescence	− 2.66
GO:0007568	GO Biological Processes	Aging	− 2.22
GO:0051092	GO Biological Processes	Positive regulation of NF-kappaB transcription factor activity	− 2.17
GO:0003007	GO Biological Processes	Heart morphogenesis	− 2.04
*10-Month-old*			
GO:0046631	GO Biological Processes	Alpha–beta T cell activation	− 4.14
GO:0050878	GO Biological Processes	Regulation of body fluid levels	− 3.54
GO:0006820	GO Biological Processes	Anion transport	− 2.48
GO:0090277	GO Biological Processes	Positive regulation of peptide hormone secretion	− 2.39
GO:0050728	GO Biological Processes	Negative regulation of inflammatory response	− 2.13

**Fig. 4:**
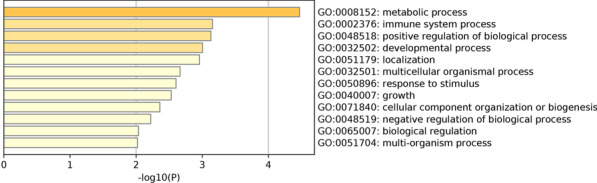
The enrichment analysis of 397 specific gene markers

The GO and KEGG pathway enrichment results for all the 397 marker genes are shown in Fig. 4. Figure 4 shows that the functions, such as metabolic process, immune system process, developmental process, growth, etc. change significantly between different disease state.

### Prediction performance of FC, t-test, DESeq2, edgeR, limma, jNMFMA, FNMF, and LPFS

**Fig. 5:**
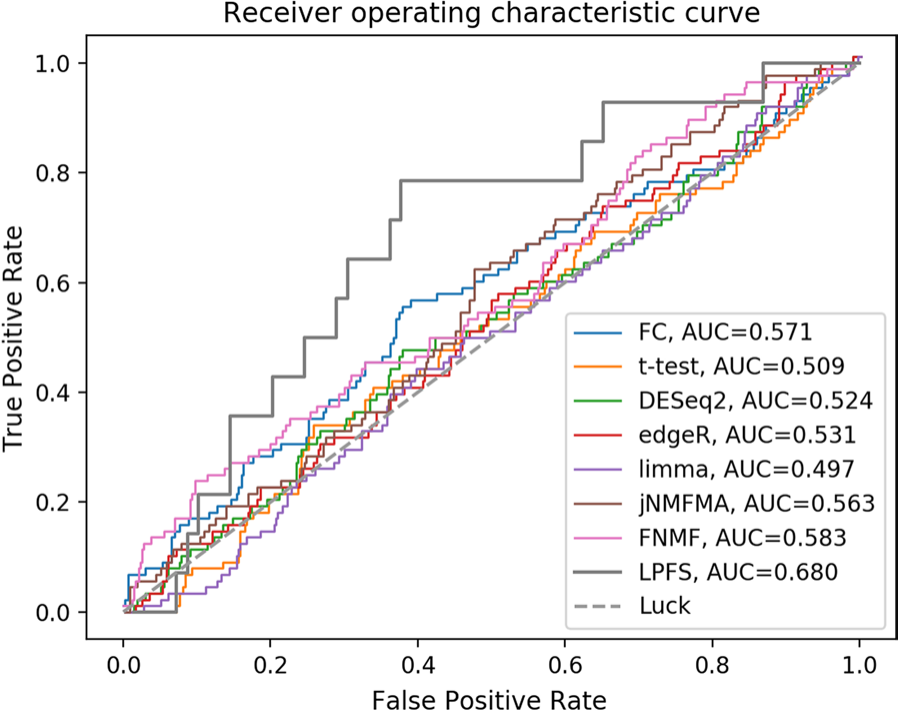
The ROC curves of FC, t-test, DESeq2, edgeR, limma, jNMFMA, and LPFS

**Fig. 6:**
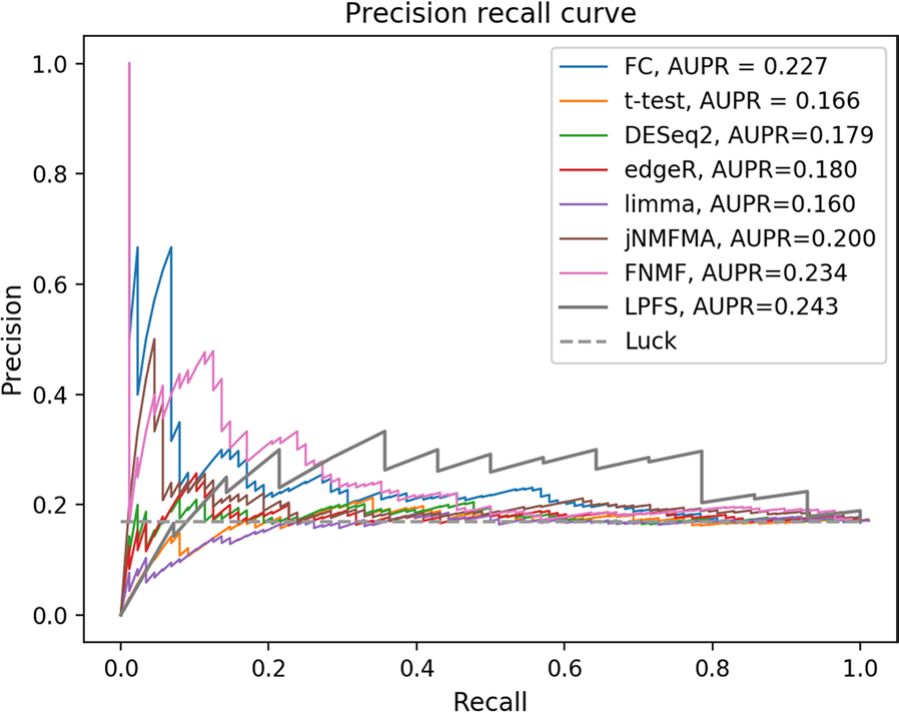
The precision recall curves of FC, t-test, DESeq2, edgeR, limma, jNMFMA, and LPFS

To verify the effectiveness of LPFS, we also conducted experiments with FC, t-test, DESeq2, edgeR, limma, jNMFMA, and FNMF. Hamming accuracy, one-error, coverage, area under ROC curve (AUC) and area under precision-recall (AUPR) curve were used as evaluative criteria of prediction accuracy. The experimental results of LPFS were shown in Table 5. The comparison results of the 8 methods were shown in Table 6, which indicates that the performance of LPFS was comparable to that of the-state-of-art methods. We further choose the best performed result of each method to draw the ROC curves and PR curves. The ROC curves and PR curves of the 8 methods were shown in Figs. 5, and 6, respectively. We could know that LPFS performs better than other methods.

**Table 5:** The performance of LPFS for disease gene selection and sample label prediction

Experiment	Hamming loss	One-error	Coverage	AUC	AUPR
Q20 versus Q80	0.210 ± 0.027	0.676 ± 0.081	0.382 ± 0.404	0.513 ± 0.064	0.193 ± 0.024
Q20 versus Q92	0.220 ± 0.021	0.707 ± 0.063	0.397 ± 0.313	0.524 ± 0.060	0.211 ± 0.024
Q20 versus Q111	0.229 ± 0.024	0.733± 0.071	0.410 ± 0.353	0.556 ± 0.058	0.186 ± 0.020
Q20 versus Q140	0.226 ± 0.016	0.724 ± 0.048	0.406 ± 0.241	0.570 ± 0.066	0.210 ± 0.031
Q20 versus Q175	0.226 ± 0.015	0.726 ± 0.046	0.407 ± 0.232	0.605 ± 0.067	0.226 ± 0.015

**Table 6:** The AUC and AUPR of different methods

Methods	FC	t-test	DESeq2	edgeR	limma	jNMFMA	FNMF	LPFS
AUC	0.570	0.509	0.524	0.531	0.497	0.547 ± 0.033	0.548 ± 0.019	0.554 ± 0.063
AUPR	0.227	0.166	0.179	0.180	0.160	0.188 ± 0.02	0.196 ± 0.01	0.205 ± 0.023

**Table 7:** The overlap degree of the top 1000 genes obtained by any two methods (397 genes for LPFS)

	DESeq2	edgeR	limma	t-test	FC	jNMFMA	FNMF
edgeR	523						
limma	312	457					
t-test	463	539	435				
FC	230	362	304	221			
jNMFMA	175	252	304	192	546		
FNMF	120	141	246	147	215	213	
LPFS	36	77	242	80	121	81	71

In addiation, we statistices the overlap degree of top 1000 genes obtained by any two methods (397 genes for LPFS). The details are shown in Table 7. Finally, we get intersection genes of the top 1000 genes obtained by the 8 methods. There are 9 overlapped genes in total, i.e., SLC4A11 (Solute Carrier Family 4 Member 11, GOTERM_BP_DIRECT: cellular cation homeostasis, fluid transport), ZFP474 (zinc finger protein 474, GOTERM_MF_DIRECT: metal ion binding), CD209G (CD209g antigen, GOTERM_MF_DIRECT: carbohydrate binding), AMBP (alpha 1 microglobulin/bikunin, GOTERM_BP_DIRECT: negative regulation of peptidase activity, protein-chromophore linkage, protein catabolic process, protein maturation), TOP2A (topoisomerase (DNA) II alpha 170kDa, GOTERM_MF_DIRECT: ATP binding, DNA binding), PBK (PDZ binding kinase, GOTERM_BP_DIRECT: negative regulation of proteasomal ubiquitin-dependent protein catabolic process, negative regulation of stress-activated MAPK cascade, cellular response to UV, negative regulation of inflammatory response), CCDC33 (coiled-coil domain containing 33, COG_ONTOLOGY: cell division and chromosome partitioning), CAPSL (calcyphosine like, GOTERM_MF_DIRECT: calcium ion binding), DLGAP5 (DLG associated protein 5, GOTERM_BP_DIRET: cell cycle, signaling), and Al662270 (have no annotation information yet), annotated with DAVID [38, 39]. The annotations of these genes indicate that the function of fluid transport, metal ion binding, the regulation of inflammatory response, cell division, cell cycle, and calcium ion binding are severally affected with the progress of the disease. Moreover, by investigating the human prefrontal cortex single cell expression files, we found that Ccdc33 mainly expressed in astrocytes and GABAergic neurons, Capsl mainly expressed in neurons and GABAergic neurons, while Dlgap5 can expressed in astrocytes, neurons, microglia, OPC, stem cells, and GABAergic neurons. This indicates that the neuron, astrocyte, microglia, GABAergic neuron, and OPC may be involved in the pathological process.

## Conclusions

Precisely decode the pathological mechanism of neurodegenerative disease is the prerequisite for the diagnosis and treatment of it. Recently, with the accumulation of omics data and clinical data, we could conduct more detailed analysis of the phenotype of the disease at different pathological stages.

In this study, to screen key genes associated with different disease stages or clinical phenotypes, we designed LPFS to screen key genes that specific identify or distinguish different disease stages. Large amounts of experiments have been conducted to investigate and verify the performance of LPFS. Then, GO and pathway enrichment analysis was been conducted to make a deep understanding of biological functions of key genes for each disease stage. Finally, by intersecting top ranking genes of the 8 methods, we found 9 novel genes, including SLC4A11, ZFP474, CD209G, TOP2A, PBK, CCDC33, CAPSL, DLGAP5, and AL662270, are seriously affected with the progressive of Huntington’s disease. Moreover, we found that the neuron, astrocyte, microglia, GABAergic neuron, and OPC could be involved in the pathological process.

## Data Availability

The gene expression data used in this study were downloaded from http://www.hdinhd.org. To make the dataset available to public, we deposit it in publicly available repository, please download at https://figshare.com/s/171c8ade2e7051556356, https://figshare.com/s/c74ac543e4893e283259, and https://figshare.com/s/ae4575a6185f6326e710. The modifier genes were from “Langfelder P, Cantle J P, Chatzopoulou D, et al. Integrated genomics and proteomics define huntingtin CAG length-dependent networks in mice. Nature Neuroscience, 2016. PMID: 26900923 DOI: 10.1038/nn.4256”. We also deposit it in publicly available repository, please download at https://figshare.com/s/13fdc5c17d736142dcd0.

## Abbreviations

FC: Fold change
FNMF: Flexible non-negative matrix factorization
jNMFMA: Joint non-negative matrix factorization meta-analysis method
ROC: Receiver operating characteristic
PR: Precision-recall
AUC: The area under the ROC curve
AUPR: The area under the PR curve

## Declarations

### Code Availability

The Python code utilized in this study has been deposited in GitHub in the repository LPFS [https://github.com/Jiang1Xue/LPFS].

## References

[1] Appel SH, Smith RG, Le WD (1996). Immune-mediated cell death in neurodegenerative disease. Adv Neurol.

[2] Hardy J (2010). Pathways to primary neurodegenerative disease. Mayo Clin Proc.

[3] Katharine G (2014). Neurodegenerative disease: brain windfall. Nature.

[4] Kaplin AI, Montel W (2007). How common are the “common” neurologic disorders?. Neurology.

[5] Martin JB (1999). Molecular basis of the neurodegenerative disorders. N Engl J Med.

[6] Browne SE, Bowling AC, Macgarvey U, Baik MJ, Berger SC, Muqit MM, Bird ED, Beal MF (2010). Oxidative damage and metabolic dysfunction in Huntington’s disease: selective vulnerability of the basal ganglia. Ann Neurol.

[7] Dobson CM (2003). Protein folding and misfolding. Nature.

[8] Lee S, Kim HJ (2015). Prion-like mechanism in amyotrophic lateral sclerosis: are protein aggregates the key?. Exp Neurobiol.

[9] Lim J, Yue Z (2015). Neuronal aggregates: formation, clearance, and spreading. Dev Cell.

[10] Ross CA, Aylward EH, Wild EJ, Langbehn DR, Tabrizi SJ (2014). Huntington disease: natural history, biomarkers and prospects for therapeutics. Nat Rev Neurol.

[11] Seredenina T, Luthi-Carter R (2012). What have we learned from gene expression profiles in Huntington’s disease?. Neurobiol Dis.

[12] Wang X, Huang T, Bu G, Xu H (2014). Dysregulation of protein trafficking in neurodegeneration. Mol Neurodegener.

[13] Difiglia M, Sapp E, Chase KO, Davies SW, Bates GP, Vonsattel JP, Aronin N (1997). Aggregation of Huntingtin in neuronal intranuclear inclusions and dystrophic neurites in brain. Science.

[14] Waldvogel HJ, Kim EH, Thu DC, Tippett LJ, Faull RL (2012). New perspectives on the neuropathology in Huntington’s disease in the human brain and its relation to symptom variation. J Huntingtons Dis.

[15] Ideker T, Ozier O, Schwikowski B, Siegel AF (2002). Discovering regulatory and signalling circuits in molecular interaction networks. Bioinformatics.

[16] Shinya Y, Manish J, Wu-Lin C, Tomasz G, Ender K, Ghayda M, Wojciech W, Hector S, Haelterman NA, Bo X (2014). A drosophila genetic resource of mutants to study mechanisms underlying human genetic diseases. Cell.

[17] Jiang X, Zhang H, Duan F, Quan X (2017). Identify Huntington’s disease associated genes based on restricted Boltzmann machine with RNA-SEQ data. BMC Bioinform.

[18] Xue J, Hang Z, Zhao Z, Quan X (2018). Flexible non-negative matrix factorization to unravel disease-related genes. IEEE/ACM Trans Comput Biol Bioinform.

[19] Jiang D, Tang C, Zhang A (2004). Cluster analysis for gene expression data: a survey. IEEE Trans Knowl Data Eng.

[20] Zhang B, Horvath S (2005). A general framework for weighted gene co-expression network analysis. Stat Appl Genet Mol Biol.

[21] Frey BJ, Delbert D (2007). Clustering by passing messages between data points. Science.

[22] Yu Z, Chen H, You J, Liu J, Wong HS, Han G, Le L (2015). Adaptive fuzzy consensus clustering framework for clustering analysis of cancer data. IEEE/ACM Trans Comput Biol Bioinform.

[23] Van Mechelen I, Bock HH, De Boeck P (2004). Two-mode clustering methods: a structured overview. Stat Methods Med Res.

[24] Cheng Y, Church GM. Biclustering of expression data. In: Eighth international conference on intelligent systems for molecular biology. 2000. 10977070

[25] Robinson MD, Smyth GK (2008). Moderated statistical tests for assessing differences in tag abundance. BMC Bioinform.

[26] Lazzeroni OAL (2002). Plaid models for gene expression data. Stat Sin.

[27] Robinson MD, Smyth GK (2008). Moderated statistical tests for assessing differences in tag abundance. Bioinformatics.

[28] Robinson MD, McCarthy DJ, Smyth GK (2010). edgeR: a bioconductor package for differential expression analysis of digital gene expression data. Bioinformatics.

[29] Ritchie ME, Belinda P, Di W, Yifang H, Law CW, Wei S, Smyth GK (2015). limma powers differential expression analyses for RNA-sequencing and microarray studies. Nucleic Acids Res.

[30] Fangxin H, Rainer B (2008). A comparison of meta-analysis methods for detecting differentially expressed genes in microarray experiments. Bioinformatics.

[31] Hong-Qiang W, Chun-Hou Z, Xing-Ming Z (2015). *j* NMFMA: a joint non-negative matrix factorization meta-analysis of transcriptomics data. Bioinformatics.

[32] Ding CHQ, Ding Z, He X, Zha H. R$$_1$$PCA: rotational invariant L$$_1$$-norm principal component analysis for robust subspace factorization. In: International conference on machine learning. 2006.

[33] Liu SMH, Fu Y. Consensus guided unsupervised feature selection. In: Proceedings of the association for the advancement of artificial intelligence, Phoenix, AZ, USA, 12–17 Feb 2016.

[34] Langfelder P, Cantle JP, Chatzopoulou D, Wang N, Gao F, Al-Ramahi I, Lu XH, Ramos EM, El-Zein K, Zhao Y (2016). Integrated genomics and proteomics define huntingtin CAG length—dependent networks in mice. Nat Neurosci.

[35] Da WH, Sherman BT, Lempicki RA (2009). Systematic and integrative analysis of large gene lists using DAVID bioinformatics resources. Nat Protoc.

[36] Wei HD, Sherman BT, Lempicki RA (2009). Bioinformatics enrichment tools: paths toward the comprehensive functional analysis of large gene lists. Nucleic Acids Res.

[37] Zhou Y, Zhou B, Pache L, Chang MW, Khodabakhshi AH, Tanaseichuk O, Benner C, Chanda SK (2019). Metascape provides a biologist-oriented resource for the analysis of systems-level datasets. Nat Commun.

[38] Huang D, Sherman BT, Lempicki RA (2009). Systematic and integrative analysis of large gene lists using DAVID bioinformatics resources. Nat Protoc.

[39] Huang D, Sherman BT, Lempicki RA (2009). Bioinformatics enrichment tools: paths toward the comprehensive functional analysis of large gene lists. Nucleic Acids Res.

